# Clinical evaluation of primary human papillomavirus (HPV) testing with extended HPV genotyping triage for cervical cancer screening: A pooled analysis of individual patient data from nine population‐based cervical cancer screening studies from China

**DOI:** 10.1002/cam4.7316

**Published:** 2024-06-03

**Authors:** Changchang Dun, Meiwen Yuan, Xuelian Zhao, Shangying Hu, Marc Arbyn, Fanghui Zhao

**Affiliations:** ^1^ Department of Population Medicine, School of Population Medicine and Public Health Chinese Academy of Medical Sciences and Peking Union Medical College Beijing China; ^2^ Department of Epidemiology, National Cancer Center/National Clinical Research Center for Cancer/Cancer Hospital Chinese Academy of Medical Sciences and Peking Union Medical College Beijing China; ^3^ Unit of Cancer Epidemiology, Belgian Cancer Centre Scientific Institute of Public Health Brussels Belgium

**Keywords:** cervical cancer, HPV genotyping, pooled analysis, screening, triage

## Abstract

**Objective:**

To assess the clinical values of extended human papillomavirus (HPV) genotyping in triage of high‐risk HPV‐positive women, focusing on the trade‐off between cervical precancer detections and colposcopy referrals.

**Methods:**

A bivariate random‐effects model was used to estimate the diagnostic accuracy of primary HPV screening with following triage strategies to detect cervical precancers: (i) partial genotyping for HPV16/18 combined with cytological testing at atypical squamous cells of undetermined significance threshold (used as the comparator), (ii) genotyping for HPV16/18/58/52, (iii) genotyping for HPV16/18/58/52/33, (iv) genotyping for HPV16/18/58/33/31, (v) genotyping for HPV16/18/58/52/33/31, and (vi) genotyping for HPV16/18/58/52/33/31/39/51. Internal risk benchmarks for clinical management were used to evaluate the risk stratification of each triage strategy.

**Results:**

A total of 16,982 women (mean age 46.1 years, range 17–69) were included in this analysis. For CIN3+ detection, triage with HPV16/18/58/33/31 genotyping achieved lower positivity (6.85% vs. 7.35%, *p* = 0.001), while maintaining similar sensitivity (91.35% vs. 96.42%, *p* = 0.32) and specificity (94.09% vs. 93.67%, *p* = 0.56) compared with the comparator strategy. Similar patterns were observed for CIN2+ detection. Women with a positive HPV16/18/58/33/31 genotyping test had high enough risk for CIN3+ for colposcopy referral, while the risk for women with a negative test was below the 1‐year return decision threshold according to internal benchmarks.

**Conclusions:**

Our findings suggested extended HPV genotyping is of potential to be used as a triage technique integrated into HPV‐based cervical cancer screening, leading to reduced need for colposcopy referral while maintaining similar disease detection and efficient risk stratification.

## 1 INTRODUCTION

The World Health Organization (WHO) has recommended human papillomavirus (HPV) testing as the first‐choice method for cervical cancer screening due to its superior clinical performance.^1^, ^2^, ^3^ However, a second triage test is typically needed to provide further risk stratification given the transient nature of most HPV infections in women. Genotyping for HPV16 and 18 is commonly used as a triage technique as an estimated 70% cervical cancers are caused by these two types.^4^, ^5^ Women positive for non‐16/18 high‐risk HPV (hrHPV) were considered to have a risk below the threshold of colposcopy referral but still not safe enough to return to routine screening,^6^, ^7^ and are therefore recommended to undergo cytological testing according to the cervical cancer screening guidelines in several countries.^8^, ^9^, ^10^ However, the roll‐out of cytology has been impeded by its demanding requirements for infrastructure and skilled healthcare personnel, particularly in low‐resource settings where the burden of cervical cancer is most severe.^11^, ^12^ Recently, extended HPV genotyping has emerged as a promising approach for risk discrimination, with accruing evidence suggesting similar or even higher predictive values for cervical precancerous lesions associated with genotypes other than HPV16/18.^13^ Meanwhile, multiple genotyping platforms are now available which allow self‐taken vaginal samples and point‐of‐care results, rendering extended HPV genotyping a more viable option. However, there is a paucity of real‐world data comparing the clinical value of extended HPV genotyping triage with the currently approved HPV16/18 partial genotyping in conjunction with cytological triage for hrHPV‐positive women in China.

Therefore, we conducted a pooled analysis using individual patient data from nine population‐based cervical cancer screening studies carried out in China. We estimated the type‐specific HPV prevalence among all participants and stratified by pathology grade to establish appropriate genotyping triage strategies. The strategy of HPV16/18 partial genotyping in conjunction with cytological (at atypical squamous cells of undetermined significance [ASC‐US] threshold) triage was used as the comparator for clinical evaluations for various HPV genotyping triage strategies regarding disease detection and risk stratification.

## 2 MATERIALS AND METHODS

### 2.1 Setting and participants

Our pooled analysis used individual patient data from nine population‐based cervical‐cancer screening studies conducted between 2005 and 2019 in two urban and five rural areas across six provinces, spanning the regions of North, Central, East, and Northwest China. Eligible women were sexually active, not pregnant, with an intact uterus, and had no history of cervical intraepithelial neoplasia (CIN), cervical cancer, or pelvic radiation. Recruitment for all studies was community‐based to minimize selection bias. Detailed information on individual studies is summarized in Table 1 and has been published in previous reports.^14^, ^15^, ^16^, ^17^ All women included in this pooled analysis underwent primary HPV screening and had reflex HPV genotyping and liquid‐based cytology (LBC) testing results. Written informed consent was obtained from all participants. The studies were approved by the Human Subjects Review Boards of Cancer Hospital Chinese Academy of Medical Sciences (CHCAMS) and other related institutions.

**TABLE 1:** Characteristics of included studies.

Study	Year; location	Number screened	Age range (years)	Primary screening tests	Follow‐up procedure	Histology or cytology location and review
SPOCCS I (follow‐up)	2005; Xiangyuan County, Shanxi Province	1766	40–52	HC2, LBC, VIA, colposcopy	Abnormality under colposcopy: directed biopsies, ECC if unsatisfactory colposcopy^a^; Positive HC2 and ASC‐US on LBC or ASC‐H or worse: second colposcopy and 4‐quadrant biopsies, ECC if unsatisfactory colposcopy^a^; Positive HC2: HPV full genotyping	CHCAMS
SPOCCS II (follow‐up)	2019; Xiangyuan and Yangcheng County, Shanxi Province	2112	49–69	HC2, LBC, p16 testing, HPV full genotyping	Positive HC2 or positive for non‐16/18 HPV types or ASC‐US on LBC: colposcopy and directed biopsies (when there were visible abnormalities)/4‐quadrant biopsies (when there were no visible abnormalities), ECC if unsatisfactory colposcopy^a^; Positive for HPV16/18 or positive p16 or ASC‐H or worse on LBC: colposcopy, 4‐quadrant biopsies, and ECC	CHCAMS
SPOCCS III‐(1)	2006; Xiangyuan County, Shanxi Province	884	16–54	HC2, LBC, VIA	Positive self‐collected HC2 or positive VIA: colposcopy and directed biopsy, ECC if unsatisfactory colposcopy^a^; Positive physician‐collected HC2 or ASC‐H or worse on LBC: colposcopy and 4‐quadrant biopsies, ECC if unsatisfactory colposcopy^a^; Positive self or physician‐collected HC2: HPV full genotyping	CHCAMS, blinded international review (only histology)
SPOCCS III‐(2)	2006; Beijing City	795	16–54	HC2, LBC, VIA	Positive VIA: colposcopy and directed biopsy, ECC if unsatisfactory colposcopy^a^; Positive physician‐collected HC2 and ASC‐US on LBC or ASC‐H or worse: colposcopy and 4‐quadrant biopsies, ECC if unsatisfactory colposcopy^a^; Positive HC2: HPV full genotyping	Peking University People's Hospital, blinded international and CHCAMS review
SPOCCS III‐(3)	2006; Xinmi County, Henan Province	879	16–54	HC2, LBC, VIA	Same as SPOCCS III‐(1)	CHCAMS, blinded international review (only histology)
SPOCCS III‐(4)	2006; Yutian County, Xinjiang Uygur Autonomous Region	883	16–54	HC2, LBC, VIA	Same as SPOCCS III‐(1)	CHCAMS, blinded international review (histology); People's Hospital of Xinjiang Uygur Autonomous Region, blinded CHCAMS review (cytology)
SPOCCS III‐(5)	2007; Shanghai City	774	16–54	HC2, LBC, VIA	Positive VIA: colposcopy and directed biopsy, ECC if unsatisfactory colposcopy^a^; Positive physician‐collected HC2 or ASC‐H or worse: colposcopy and four‐quadrant biopsies, ECC if unsatisfactory colposcopy^a^; Positive HC2: HPV full genotyping	Shanghai, blinded international and CHCAMS review (for cytology, blinded CHCAMS review only)
POCST study‐(1)	2017; Etuoke County, Inner Mongolia Autonomous Region	2398	33–65	*care*HPV, Sansure HPV assay	Positive *care*HPV or Sansure HPV assay: LBC, colposcopy and directed biopsy (when there were visible abnormalities)/4‐quadrant biopsies (when there were no visible abnormalities), ECC if unsatisfactory colposcopy^a^; and HPV full genotyping	CHCAMS
POCST study‐(2)	2017; Xiangyuan County and Yangcheng County, Shanxi Province	7128	29–65	*care*HPV, Sansure HPV assay	Same as POCST study‐(1)	CHCAMS

Abbreviations: ASC‐H, atypical squamous cells cannot exclude high‐grade squamous intraepithelial lesion; ASC‐US, atypical squamous cells of undetermined significance; CHCAMS, Cancer Hospital Chinese Academy of Medical Sciences; ECC, endocervical curettage; HC2, Hybrid Capture 2; HPV, human papillomavirus; LBC, liquid‐based cytology; PCR, polymerase chain reaction; POCST study, Point of Care Screening and Treatment for Cervical Cancer Study; SPOCCS, Shanxi Province Cervical Cancer Screening Study; VIA, visual inspection with acetic acid.

^a^ An unsatisfactory colposcopy was determined when the cervical squamo‐columnar junction was not fully visible.

### 2.2 Study procedures

#### 2.2.1 hrHPV testing

In the Shanxi Province Cervical Cancer Screening Study (SPOCCS), HPV testing was performed in CHCAMS central laboratory with Hybrid Capture 2 High‐Risk HPV DNA test (Qiagen, Gaithersburg, MD, USA) which detects 13 hrHPV types of 16, 18, 31, 33, 35, 39, 45, 51, 52, 56, 58, 59, and 68 in aggregate.^18^ In the Point of Care Screening and Treatment for Cervical Cancer Study (POCST study), the *care*HPV (Qiagen, Shenzhen, China) and polymerase chain reaction (PCR)‐based Sansure HPV assay (Sansure, Changsha, China) were used by trained laboratory technicians at local maternity and children's hospitals or family planning service centers. The *care*HPV test identifies in aggregate 14 hrHPV types (HPV16, 18, 31, 33, 35, 39, 45, 51, 52, 56, 58, 59, 66, and 68), and the PCR‐based Sansure HPV assay identifies in aggregate 15 hrHPV types (HPV16, 18, 31, 33, 35, 39, 45, 51, 52, 53, 56, 58, 59, 66, and 68).^19^ All HPV testing was conducted following the manufacturer's instructions.

#### 2.2.2 Cytology

All women in the SPOCCS and all HPV‐positive women in the POCST study had LBC (ThinPrep, Hologic, Bedford, MA, USA or SurePath, BD Diagnostics, Franklin Lakes, NJ, USA) results. All cytological slides were examined at CHCAMS except for SPOCCS III‐Xinjiang where slides were evaluated by on‐site pathologists and reviewed by senior pathologists at CHCAMS. The cytological results were interpreted by using the Bethesda terminology.^20^

#### 2.2.3 HPV genotyping

HPV‐positive samples were subjected to genotyping by various assays across projects. In SPOCCS I‐2005, the INNO‐LiPA HPV genotyping Extra assay (Innogenetics NV, Gent Belgium; Innogenetics now renamed as Fujirebio Europe) which identifies individually 28 HPV genotypes was used.^21^ In SPOCCS II‐2019, the SureX HPV 25X Genotyping Kit (Health Gene Tech, Ningbo, China) which identifies individually 25 HPV genotypes was used.^15^ In SPOCCS III, we used the Linear Array HPV genotyping test (Roche, Pleasanton, CA, USA) identifying 37 HPV genotypes individually.^22^ In the POCST study, we used the Sansure HPV genotyping kit (Sansure, Changsha, China) identifying 15 HPV genotypes individually.^17^ The characteristics of each HPV genotyping assay used in the included studies are listed in Table S1. In SPOCCS HPV genotyping was performed at CHCAMS laboratory and in POCST study HPV genotyping was performed at the local laboratory by trained technicians following the manufacturer's instructions.

#### 2.2.4 Biopsy and verification of disease status

In SPOCCS I‐2005 all women received colposcopy and in the other studies, women who tested positive for any primary screening underwent colposcopy examinations. Directed biopsy was performed in the presence of colposcopically visible lesions. Endocervical curettage (ECC) was performed when the colposcopy examinations were unsatisfactory (the squamous‐columnar junction was not completely visible), whereas in SPOCCS II‐2019 all women with positive HPV16/18 or p16, or had low‐grade squamous intraepithelial lesion (LSIL) or worse on cytology additionally received ECC due to the older age at screening. When the four‐quadrant punch biopsy method was indicated, random biopsies were taken at the 2, 4, 8, and 10 o'clock positions or 3, 6, 9, and 12 o'clock positions. Criteria for four‐quadrant random biopsy in the included studies are shown in Table 1.

To pool the data, we established uniform criteria for disease ascertainment. Disease status was classified as negative, CIN grade 1 (CIN1), 2 (CIN2), or 3 (CIN3), squamous cell carcinoma (SCC), adenocarcinoma in situ (AIS), and adenocarcinoma (ADC). Histological diagnoses from biopsy served as the gold standard for ascertainment of the disease outcome. Women without biopsy but meeting one of the following conditions were considered disease‐free given the extremely low risk of high‐grade cervical neoplasia based on previous studies^16^, ^23^, ^24^, ^25^: (i) negative for all primary screening tests; (ii) negative or ASC‐US on cytology and negative for hrHPV; (iii) negative on cytology, positive for hrHPV but negative for colposcopy. Women without biopsy but falling in any of the following categories were considered as having incomplete data for disease status and were therefore excluded from this analysis^16^: (i) ASC‐US on cytology and hrHPV‐positive; (ii) LSIL or worse cytology; (iii) hrHPV‐positive, negative cytology, and missing or positive colposcopy; (iv) unsatisfactory or no cytology results. Most biopsies were processed and interpreted at CHCAMS except those in SPOCCS III‐Beijing and SPOCCS III‐Shanghai were interpreted by local pathologists. Furthermore, biopsy results from five studies were reviewed by international expert pathologists for quality control.

### 2.3 Statistical analysis

The primary outcome was the diagnostic absolute and relative accuracy of HPV primary screening with various triage testing for colposcopy referral to detect CIN3 or higher (CIN3+). The type‐specific prevalence for 14 individual hrHPV genotypes (HPV16, 18, 31, 33, 35, 39, 45, 51, 52, 56, 58, 59, 66, and 68) among all participants and stratified by pathology grade (normal, CIN1, CIN2, and CIN3+) were calculated. HPV genotyping triage strategies were developed by grouping HPV genotypes based on their prevalence in CIN3+. The following six triage strategies were examined: (i) partial genotyping for HPV16/18 combined with cytological testing at ASC‐US threshold (used as the comparator), (ii) genotyping for HPV16/18/58/52, (iii) genotyping for HPV16/18/58/52/33, (iv) genotyping for HPV16/18/58/33/31, (v) genotyping for HPV16/18/58/52/33/31, and (vi) genotyping for HPV16/18/58/52/33/31/39/51. The pooled sensitivity and specificity for each strategy were calculated using bivariate random‐effects model as recommended by the Cochrane Collaboration.^26^ Summary receiver‐operating characteristic curves (SROC) and forest plots were used to display study‐specific and pooled accuracy estimates. The relative sensitivity and specificity were calculated by adding the comparator strategy as a covariate into the bivariate model.^27^ Differences in positivity rate were assessed with McNemar *χ* ^2^ test. In a supplemental analysis, we stratified diagnostic performance metrics by age groups (≤45 and >45 years).

The secondary outcome was the risk stratification of each triage strategy measured by posttest risks for CIN3+. We calculated the posttest risks based on the average prevalence of cervical precancers in strata by different combinations of the assessed testing results.^28^ We plotted posttest risks against internal benchmark levels derived from the current Chinese management guidelines^29^: the risk of precancers in HPV‐positive women with ASC‐US in the observed population (2.24%) was the threshold for referral to colposcopy; the risk of precancers in HPV‐positive women and negative for intraepithelial lesion or malignancy (NILM, 0.54%) was the threshold for 1‐year return.

All analyses were performed with “metadta” modules in StataMP (version 17) and “lme4” package in R (version 4.1.3).^30^, ^31^ All statistical tests were two‐sided, and a 0.05 level of significance was applied.

## 3 RESULTS

### 3.1 Study population

**FIGURE 1:**
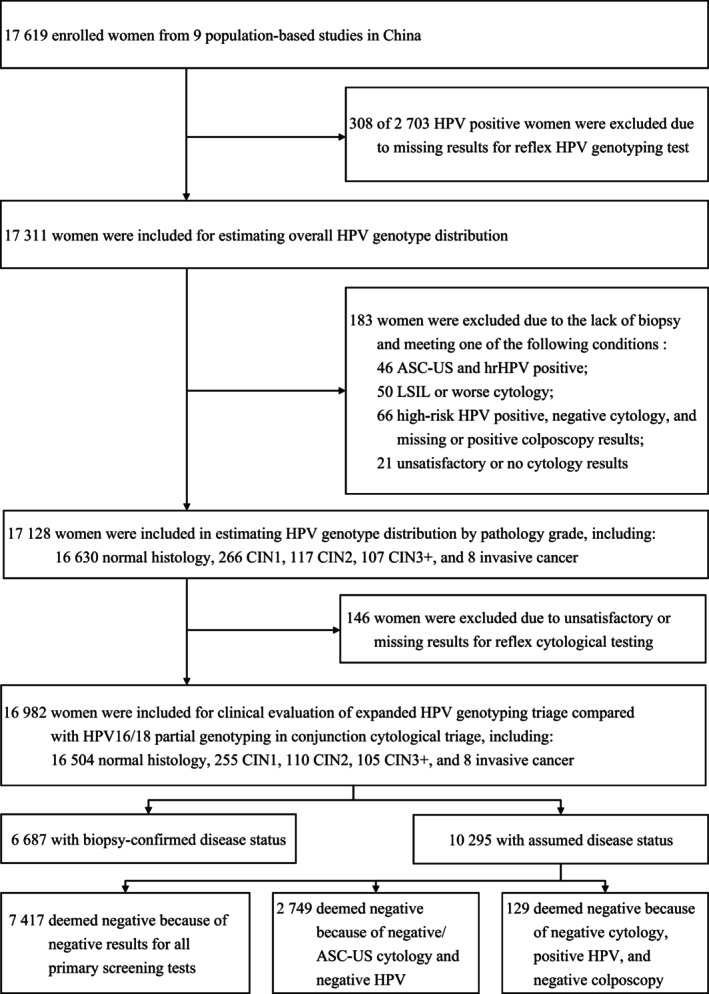
Flow diagram of participant selection.

The flow diagram of participant selection is shown in Figure 1. Among 17,619 eligible women enrolled in nine studies, 2703 (15.34%) tested positive for hrHPV at primary screening. Of these, 308 had missing results for reflex HPV genotyping and therefore 17,311 women were included for estimating HPV type‐specific prevalence. To evaluate HPV genotype distribution across pathology grades, 183 women were additionally ruled out due to incomplete disease ascertainment. Of the remaining 17,128 women, 146 women were further excluded due to unsatisfactory or missing reflex cytological results. Thus, 16,982 women with valid results for HPV genotyping and cytology and ascertained disease status were included for clinical evaluations for different strategies, of whom 6687 had biopsy‐confirmed (39.39%) and 10,295 had assumed (60.61%) final diagnosis. The mean age of the 16,982 participants was 46.12 [standard deviation (SD) 9.86; range 17–69]. Menstrual data were available for 16,964 (99.89%) women and 5104 were confirmed postmenopausal, with an estimated mean menopausal age of 49.67 (SD 4.31). Almost all women were from rural areas (90.97%; 15,449 women), with 1533 from urban areas. Of the 16,982 women, 16,504 (97.19%), 255 (1.50%), 110 (0.65%), 105 (0.62%), and 8 (0.05%) were assessed as healthy, CIN1, CIN2, CIN3, and invasive cancer, respectively.

### 3.2 Overall HPV genotype distribution and stratified by pathology grade

**FIGURE 2:**
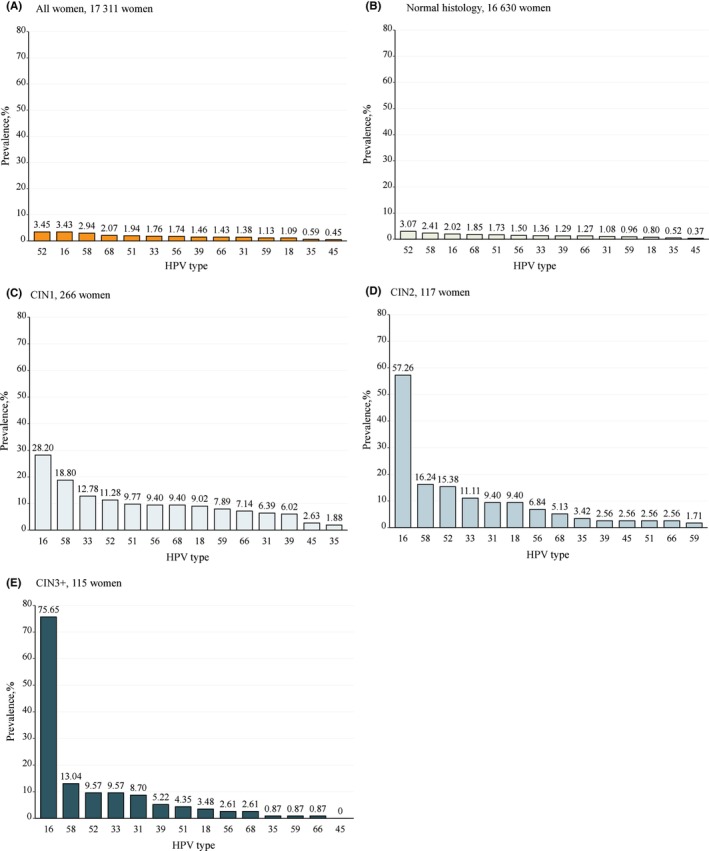
HPV genotype distribution among all women (A) and stratified by pathology grade (B) for women with normal histology, (C) for women with CIN1, (D) for women with CIN2, and (E) for women with CIN3+. CIN1, cervical intraepithelial neoplasia grade 1; CIN2, cervical intraepithelial neoplasia grade 2; CIN3+, cervical intraepithelial neoplasia grade 3 or higher; HPV, human papillomavirus.

Figure 2 presents the HPV type‐specific prevalence among all women and stratified by pathology grade. Overall, the five most common HPV genotypes were HPV52 (3.45%), HPV16 (3.43%), HPV58 (2.94%), HPV68 (2.07%), and HPV51 (1.94%). In the normal histology group, the five most prevalent HPV genotypes were HPV52 (3.07%), HPV58 (2.41%), HPV16 (2.02%), HPV68 (1.85%), and HPV51 (1.73%). In the CIN1 group, HPV16 was the most prevalent genotype (28.20%), followed by HPV58 (18.80%), HPV33 (12.78%), HPV52 (11.28%), and HPV51 (9.77%). The five most prevalent HPV genotypes were identical in the CIN2 and CIN3+ groups, although with a slight difference in their ranking order: HPV16 was the leading prevalent genotype in both pathological categories (57.26% and 75.65%, respectively), followed by HPV58 (16.24%), HPV52 (15.38%), HPV33 (11.11%), and HPV31 (9.40%) in CIN2, and HPV58 (13.04%), HPV52 (9.57%), HPV33 (9.57%, same as HPV52), and HPV31 (8.70%) in CIN3 + .

### 3.3 Diagnostic performance of primary HPV screening with various triage strategies to detect cervical precancerous lesions

**FIGURE 3:**
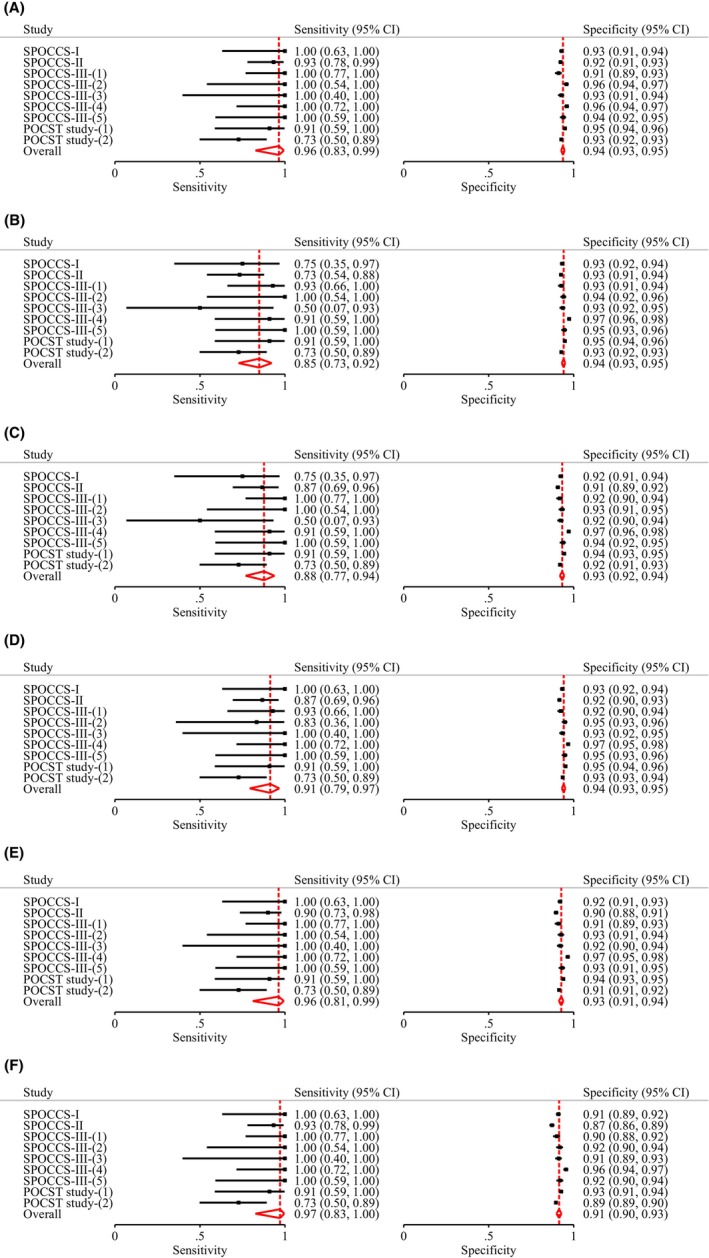
Forest plots of the sensitivity and specificity of primary HPV screening with various triage strategies for CIN3+ detection. (A) Forest plot of the sensitivity and specificity of primary HPV screening with HPV16/18 genotyping combined with cytological triage for CIN3+ detection. (B) Forest plot of the sensitivity and specificity of primary HPV screening with HPV16/18/58/52 genotyping triage for CIN3+ detection. (C) Forest plot of the sensitivity and specificity of primary HPV screening with HPV16/18/58/52/33 genotyping triage for CIN3+ detection. (D) Forest plot of the sensitivity and specificity of primary HPV screening with HPV16/18/58/33/31 genotyping triage for CIN3+ detection. (E) Forest plot of the sensitivity and specificity of primary HPV screening with HPV16/18/58/52/33/31 genotyping triage for CIN3+ detection. (F) Forest plot of the sensitivity and specificity of primary HPV screening with HPV16/18/58/52/33/31/39/51 genotyping triage for CIN3+ detection. The diamonds indicate pooled sensitivity or specificity from the bivariate random‐effects model. CI, confidence interval; CIN3+, cervical intraepithelial neoplasia grade 3 or higher; HPV, human papillomavirus; POCST study, Point of Care Screening and Treatment for Cervical Cancer Study; SPOCCS, Shanxi Province Cervical Cancer Screening Study.

**FIGURE 4:**
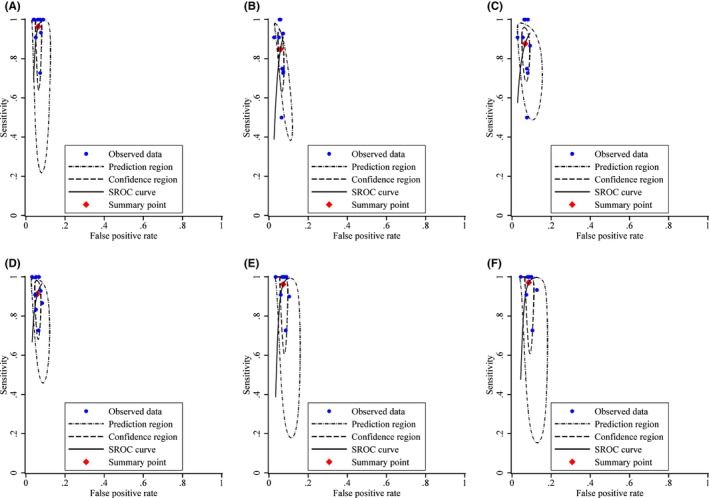
Summary of receiver operating characteristic (SROC) curves for primary HPV screening with various triage strategies for CIN3+ detection. (A) SROC curve for primary HPV screening with HPV16/18 genotyping combined with cytological triage for CIN3+ detection. (B) SROC curve for primary HPV screening with HPV16/18/58/52 genotyping triage for CIN3+ detection. (C) SROC curve for primary HPV screening with HPV16/18/58/52/33 genotyping triage for CIN3+ detection. (D) SROC curve for primary HPV screening with HPV16/18/58/33/31 genotyping triage for CIN3+ detection. (E) SROC curve for primary HPV screening with HPV16/18/58/52/33/31 genotyping triage for CIN3+ detection. (F) SROC curve for primary HPV screening with HPV16/18/58/52/33/31/39/51 genotyping triage for CIN3+ detection. CIN3+, cervical intraepithelial neoplasia grade 3 or higher; HPV, human papillomavirus.

Table 2 shows the diagnostic performance of various strategies, as well as the ratios of sensitivity and specificity for primary HPV screening with extended HPV genotyping triage versus the current HPV16/18 genotyping in conjunction with cytological triage (comparator strategy). The sensitivity of the comparator strategy for CIN3+ detection was 96.42% [95% confidence interval (CI): 82.62%–99.35%], with specificity 93.67% (95% CI: 92.58%–94.61%). As more HPV genotypes were added from the HPV16/18/58/52 genotyping, the sensitivity increased and specificity decreased, although the differences were not statistically significant except for the HPV16/18/58/52/33/31/39/51 genotyping which had a substantial lower specificity than the comparator strategy (91.40% vs. 93.67%, relative ratio 0.98 [0.96–0.99], *p* = 0.02). The positivity rate of genotyping for HPV16/18/58/52 was similar to the comparator strategy (7.05% vs. 7.35%, *p* = 0.06), whereas the positivity rate of genotyping for HPV16/18/58/33/31 (6.85%) was significantly lower (*p* = 0.001) and that for HPV16/18/58/52/33 (7.97%), HPV16/18/58/52/33/31 (8.57%), or HPV16/18/58/52/33/31/39/51 (10.12%) was significantly higher (*p* < 0.001). Figure 3 shows the forest plot and Figure 4 shows the SROC for each strategy for CIN3+ detection. Similar patterns were observed with CIN grade 2 or higher (CIN2+) endpoint (Table 1 and Figures S1 and S2). These results were consistent across all age groups (Tables S2 and S3).

**TABLE 2:** Diagnostic performance of primary HPV screening with various triage strategies for CIN3+ and CIN2+ detection.

Triage strategies	Positivity% (no./total no.)	*p* value	Pooled value% (95% CI)		Relative ratio (95% CI)			
			Sensitivity	Specificity	Sensitivity ratio	*p* value	Specificity ratio	*p* value
Detection of CIN3+ (*n* = 113)								
HPV16/18 genotyping in conjunction with cytology (ASC‐US threshold)	7.35 (1248/16,982)	Ref.	96.42 (82.62–99.35)	93.67 (92.58–94.61)	Ref.	NA	Ref.	NA
HPV16/18/58/52 genotyping	7.05 (1197/16,982)	0.06	84.87 (72.67–92.21)	94.04 (92.99–94.95)	0.88 (0.77–1.00)	0.05	1.00 (0.99–1.02)	0.60
HPV16/18/58/52/33 genotyping	7.97 (1353/16,982)	<0.001	87.68 (76.85–93.85)	93.22 (91.83–94.39)	0.91 (0.81–1.02)	0.11	1.00 (0.98–1.01)	0.59
HPV16/18/58/33/31 genotyping	6.85 (1163/16,982)	0.001	91.35 (79.26–96.69)	94.09 (93.02, 95.01)	0.95 (0.85–1.05)	0.32	1.00 (0.99–1.02)	0.56
HPV16/18/58/52/33/31 genotyping	8.57 (1456/16,982)	<0.001	96.24 (81.06–99.35)	92.67 (91.27–93.87)	1.00 (0.91–1.10)	0.97	0.99 (0.97–1.01)	0.24
HPV16/18/58/52/33/31/39/51 genotyping	10.12 (1718/16,982)	<0.001	97.12 (82.60–99.58)	91.40 (89.83–92.75)	1.01 (0.92–1.10)	0.88	0.98 (0.96–0.99)	0.02
Detection of CIN2+ (*n* = 223)								
HPV16/18 genotyping in conjunction with cytology (ASC‐US threshold)	7.35 (1248/16,982)	Ref.	94.13 (83.98–98.00)	94.18 (93.19–95.03)	Ref.	NA	Ref.	NA
HPV16/18/58/52 genotyping	7.05 (1197/16,982)	0.06	79.09 (71.82–84.88)	94.51 (93.49–95.37)	0.84 (0.76–0.93)	0.01	1.00 (0.99–1.02)	0.63
HPV16/18/58/52/33 genotyping	7.97 (1353/16,982)	<0.001	82.26 (76.23–87.03)	93.70 (92.37–94.81)	0.87 (0.80–0.96)	0.02	0.99 (0.98–1.01)	0.54
HPV16/18/58/33/31 genotyping	6.85 (1163/16,982)	0.001	84.83 (76.73–90.46)	94.57 (93.59–95.41)	0.90 (0.81–1.00)	0.06	1.00 (0.99–1.02)	0.56
HPV16/18/58/52/33/31 genotyping	8.57 (1456/16,982)	<0.001	87.54 (79.39–92.76)	93.13 (91.79–94.26)	0.93 (0.84–1.03)	0.17	0.99 (0.97–1.01)	0.19
HPV16/18/58/52/33/31/39/51 genotyping	10.12 (1718/16,982)	<0.001	90.72 (81.64–95.56)	91.92 (90.38–93.23)	0.96 (0.87–1.06)	0.46	0.98 (0.96–0.99)	0.01

Abbreviations: AUSROC, areas under the receiver operating characteristic curves; CIN3+, cervical intraepithelial neoplasia grade 3 or higher; ASC‐US, atypical squamous cells of undetermined significance; CIN2+, cervical intraepithelial neoplasia grade 2 or higher; NA not available.

### 3.4 Risk stratification of various triage strategies against internal risk benchmarks

**FIGURE 5:**
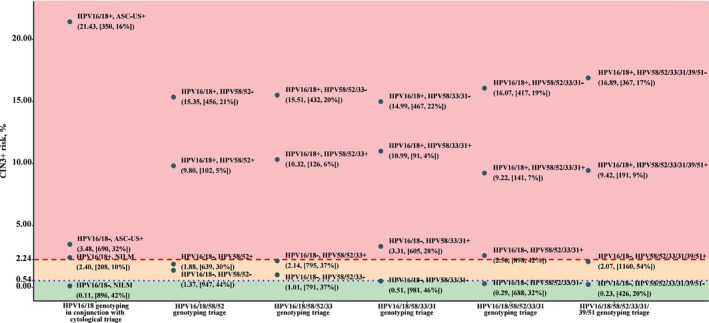
Risk of CIN3+ against internal risk benchmarks according to various triage testing results among high‐risk HPV‐positive women. The risk of CIN3+ for combinations of HPV16/18 partial genotyping with cytological testing and expanded HPV genotyping alone is plotted on the y‐axis and indicated in parentheses. The number and corresponding percentage of women for specific testing result is shown in square brackets. The dotted blue line indicated the 1‐year return threshold (HPV‐positive and negative for intraepithelial lesion or malignancy [NILM], 0.54%). The dashed red line indicated the colposcopy referral threshold (HPV‐positive and atypical squamous cells of undetermined significance [ASC‐US], 2.24%). Women with risk falling into the red zone were identified as needing immediate colposcopy referral. Women with risk falling into the yellow zone were identified as needing 1‐year return testing. Women with risk falling into the green zone were identified as needing conservative surveillance. CIN3+, cervical intraepithelial neoplasia grade 3 or higher; HPV, human papillomavirus.

Figure 5 shows CIN3+ risks among hrHPV‐positive women for different combinations of cytological testing, HPV16/18 partial genotyping, and extended HPV genotyping against internal benchmark levels. For HPV16/18 positive women, the CIN3+ risk was clearly over the colposcopy referral threshold. For non‐16/18 hrHPV‐positive women, the additional genotyping for HPV58/52 or HPV58/52/33 failed to guide clinical management as the posttest CIN3+ risk fell between the colposcopy threshold and 1‐year return threshold irrespective of testing results (in the yellow zone). The strategy of HPV16/18/58/33/31 genotyping achieved a satisfactory risk stratification with no women in yellow zone and 54% of women referred to immediate colposcopy, whereas with the current HPV16/18 genotyping combined with cytological triage 58% referred to immediate colposcopy (*p* for difference = 0.001). The strategy of HPV16/18/58/52/33/31 genotyping provided comparable risk stratification, but more women were identified as having a CIN3+ risk above the threshold for colposcopy referral (68% vs. 58% using comparator strategy, *p* < 0.001). Using HPV16/18/58/52/33/31/39/51 genotyping for triage led to a compromised risk stratification which was unable to identify those needing colposcopy referral among non‐16/18 hrHPV‐positive women. Similar patterns were observed with CIN2+ endpoint (Figure S3).

## 4 DISCUSSION

The type‐specific prevalence and etiological risk fraction of HPV have profound impacts on the development of cervical cancer prevention efforts, for both screening and vaccination. It has been established that HPV genotype distribution varied greatly by geographic region, with HPV‐16 retaining the most common type globally but HPV52 and 58 being more prevalent in Eastern Asia.^32^, ^33^ Previous studies have investigated the HPV genotype distribution among Chinese women but most were based on a single center or healthcare institutions, limiting their ability to provide representation of general population.^9^, ^29^, ^34^, ^35^, ^36^, ^37^, ^38^ In this study, we used pooled data from nine population‐based cervical cancer screening studies from six parts of China and identified that the five most frequently detected genotypes were HPV52, 16, 58, 68, and 51. We further evaluated HPV genotype distribution stratified by pathology grade as a proxy for risk assessment to identify genotypes that warrant distinct management.^33^ CIN3+ was chosen as the primary endpoint rather than CIN2+ because the diagnosis of CIN2 was found to be less histological reproducible.^39^ We found that although HPV52 is the highest prevalent genotype among all participants, HPV16 and 58 were more detected and HPV33 were equally detected in CIN3+ cases, consistent with the findings from earlier cohort studies indicating a relatively higher long‐term CIN3+ risk conferred by HPV16, 58, and 33 in Chinese women.^40^, ^41^

Several assays have been proposed to be used to triage hrHPV‐positive women for immediate colposcopy.^2^ In this study, we evaluated the triage testing together with primary HPV screening as an integrated strategy to produce estimates for diagnostic performance because the effectiveness and efficiency of a screening algorithm were associated with the combination of component results.^8^ Consequently, the specificity reported here was noticeably higher than that in studies focusing exclusively on HPV‐positive women, as a substantial number of HPV‐negative individuals were included into calculations.^42^ The comparator strategy in this study, that is, HPV16/18 partial genotyping in conjunction with cytology at ASC‐US cutoff, yielded a sensitivity (96.42%) for CIN3+ higher than most other settings. For example, the sensitivity was 78.2% in ATHENA trial and 88.2% in FRIDA study.^43^, ^44^ This difference might be attributed to the rigorous cytology evaluation in our screening studies, supported by a similar sensitivity observed in KPNC study (92.8%) where cytological testing was also performed under strict control.^8^ This also suggested that the presented superior performance of HPV genotyping triage in this study might be more evident in settings where the diagnostic accuracy of cytology cannot be guaranteed. Another possible reason for this difference could be that 183 women with abnormal primary testing results were excluded due to the lack of biopsy but in routine they were at a relatively higher risk for cervical precancerous lesions and needed triage. Nevertheless, the certain number of cervical precancer cases among these 183 women would be small and could hardly have impact on the conclusion of this study.

Determining which and how many HPV genotypes to be included into the triage strategy to achieve a balance between sensitivity, specificity, and positivity rate (indicating the number of women to be referred for colposcopy) is necessary. Based on the principle of “equal management for equal risk”, we evaluated the absolute risk of cervical precancers in women positive and negative for tirage testing against internal risk thresholds to identify what management is warranted.^45^ We identified that genotyping for HPV16/18/58/33/31 to triage HPV‐positive women is of promising use, maintaining equivalent sensitivity and specificity and offering a more efficient risk stratification (more women classified as having low CIN3+ risk below the threshold of 1‐year return and the remaining women as needing referral to undergo colposcopy examination) compared with the comparator strategy. More Specifically, the lower positive rate of HPV16/18/58/33/31 genotyping strategy compared with the reference strategy was associated with a slight but not significant loss in sensitivity for CIN3+ whereas the specificity was similar. Our findings also suggested that there might be little extra benefit to further extending the genotype panel as including more types hardly achieve statistically significant gain in sensitivity but substantially reduced specificity and increased colposcopy referral.

This study has several strengths. To our knowledge, this was the first study to estimate the type‐specific HPV prevalence and evaluate the clinical values for extended HPV genotyping compared with HPV16/18 partial genotyping in conjunction with cytology as triage for HPV‐positive women, using data from population‐based cervical cancer screening studies in China. With the global scale‐up of HPV vaccination, the clinical relevance of extended HPV genotyping will increase as the prevalence of HPV‐16/18 decreases and non‐16/18 hrHPV genotypes may play a relatively larger role in cervical disease. Findings from this study may not only provide a potential alternative strategy for managing hrHPV‐positive women but also offer insights that can inform practical recommendations for cervical cancer prevention in the post‐vaccine era.

Several limitations of this study should be acknowledged. First, the HPV genotyping triage strategies developed in this study were dependent on the HPV genotype distribution among cervical precancers, which can be heterogeneous across regions. However, it is easy for other settings to formulate tailored genotyping strategies based on their type‐specific HPV prevalence and conduct evaluations accordingly. Second, whether HPV45, a genotype associated with increased risk for ADC, should be included in the genotyping panel has been a matter of debate over time. However, we cannot assess the added value of HPV45 genotyping in this study because none of the 115 CIN3+ women were HPV45 positive and none of them were identified as AIS or ADC, which was consistent with the fact that cervical ADC is relatively rare in China. In this study we found that adding HPV45 into the genotyping panel would lead to 67 more colposcopy referral but no additional yield for CIN3+ detection, and such was not required to reach good clinical performance. However this might not be applicable in other settings where ADC is more prevalent and the benefit of HPV45 genotyping might become observable in those populations. Third, biopsies were not collected from all participants due to ethical constraints, which could have induced verification bias. In this study, we adjudicated participants as disease‐free based on multiple screening tests, and four‐quadrant punch biopsy was performed when no colposcopically suspected lesions were seen, thereby decreasing the likelihood of verification bias.

## 5 CONCLUSION

This study investigated the HPV genotype distribution in Chinese women with CIN2 and CIN3+ and found that HPV16, 58, 52, 33, and 31 were the most commonly detected genotypes. Findings from this study also suggested that extended HPV genotyping could be an alternative triage technique following HPV primary testing, which could reduce the need for colposcopy referral while maintaining similar cervical precancer detections and efficient risk stratifications. This is particularly relevant for low‐resource settings where cytological triage can be hardly applied, and could also contribute to the integration of HPV self‐sampling into cervical cancer screening.

## AUTHOR CONTRIBUTIONS

**Fanghui Zhao:** Conceptualization (lead); funding acquisition (lead); project administration (lead); supervision (equal); writing – review and editing (equal). **Changchang Dun:** Conceptualization (equal); data curation (lead); formal analysis (lead); visualization (lead); writing – original draft (lead); writing – review and editing (lead). **Meiwen Yuan:** Data curation (supporting); writing – review and editing (supporting). **Xuelian Zhao:** Conceptualization (supporting); project administration (equal); supervision (equal); writing – review and editing (supporting). **Shangying Hu:** Conceptualization (equal); project administration (equal); supervision (equal); writing – review and editing (equal). **Marc Arbyn:** Formal analysis (supporting); writing – review and editing (equal).

## FUNDING INFORMATION

This study was supported by the Chinese Academy of Medical Sciences Initiative for Innovative Medicine [grant number 2021‐I2M‐1‐004].

## CONFLICT OF INTEREST STATEMENT

Marc Arbyn reported that financial support was provided by Horizon 2020 Framework Programme for Research and Innovation of the European Commission through the RISCC Network. Other authors declared that they have no known competing financial interests or personal relationships that could have appeared to influence the work reported in this paper.

## Supporting information


**Data S1:** Supporting Information.

## Data Availability

The data that support the findings of our study are available on request from the corresponding author. The data are not publicly available due to privacy or ethical restrictions.
