# Correction to “Clinical evaluation of primary human papillomavirus (HPV) testing with extended HPV genotyping triage for cervical cancer screening: A pooled analysis of individual patient data from nine population‐based cervical cancer screening studies from China”

**DOI:** 10.1002/cam4.7451

**Published:** 2024-07-05

Dun C, Yuan M, Zhao X, Hu S, Arbyn M, Zhao F. Clinical evaluation of primary human papillomavirus (HPV) testing with extended HPV genotyping triage for cervical cancer screening: a pooled analysis of individual patient data from nine population‐based cervical cancer screening studies from China. *Cancer Med*. 2024;13:e7316. doi:10.1002/cam4.7316

Grant No. 847845 has been added to the first sentence of the CONFLICT OF INTEREST section. The sentence should read:

Marc Arbyn reported that financial support was provided by Horizon 2020 Framework Programme for Research and Innovation of the European Commission through the RISCC Network (Grant No. 847845).

