# Supplementary material for: Clinical evaluation of primary human papillomavirus (HPV) testing with extended HPV genotyping triage for cervical cancer screening: A pooled analysis of individual patient data from nine population‐based cervical cancer screening studies from China
Source: Cancer Med. 2024 Jun 3;13(11):e7316. doi: 10.1002/cam4.7316 (PMC11145129; doi:10.1002/cam4.7316)
Supplement: Supplementary file 1 — Data S1: Supporting Information. [file CAM4-13-e7316-s001.docx]

**Supplementary Online Content**

**Supplementary Table S1.** Characteristics of HPV genotyping assays used in the included studies.

**Supplementary Figure S1.** Forest plots of the sensitivity and specificity of primary HPV screening with various triage strategies for CIN2+ detection.

**Supplementary Figure S2.** Summary of receiver operating characteristic (SROC) curves for primary HPV screening with various triage strategies for CIN2+ detection.

**Supplementary Table S2.** Diagnostic performance of primary HPV screening with various triage strategies for CIN3+ and CIN2+ detection among women aged ≤45.

**Supplementary Table S3.** Diagnostic performance of primary HPV screening with various triage strategies for CIN3+ and CIN2+ detection among women aged >45**.**

**Supplementary Figure S3.** Risk of CIN2+ against internal risk benchmarks according to various triage testing results among high-risk HPV-positive women.

**Supplementary Table S1. Characteristics of HPV genotyping assays used in the included studies**

| **Study** | **HPV genotyping assay** | **Nucleic acid targeted** | **Type of amplification** | **Genes targeted** | **Separate genotyping** | **Internal control for human genes** | **Manufacturer** |
| --- | --- | --- | --- | --- | --- | --- | --- |
| SPOCCS Ⅰ (follow-up) | INNO-LiPA Extra Assay | DNA | Target (PCR) | L1 | Separate identification for 28 HPV genotypes: HPV6, 11, 16, 18, 26, 31, 33, 35, 39, 40, 43, 44, 45, 51, 52, 53, 54, 56, 58, 59, 66, 68, 69, 70, 71, 73, 74, 82) | HLA-DPB1 | Innogenetics NV, Gent Belgium; Innogenetics now renamed as Fujirebio Europe |
| SPOCCS Ⅱ (follow-up) | SureX HPV 25X Genotyping Kit | DNA | Target (PCR) | E6/E7/E1 | Separate identification for 25 HPV genotypes: HPV6, 11, 16, 18, 26, 31, 33, 35, 39, 42, 43, 44, 45, 51, 52, 53, 56, 58, 59, 66, 68, 73, 81, 82, 83) | β-globin | Health Gene Tech, Ningbo, China |
| SPOCCS III-(1) | Linear Array HPV Genotyping Test | DNA | Target (PCR) | L1 | Separate identification for 37 HPV genotypes: HPV6, 11, 16, 18, 26, 31, 33, 35, 39, 40, 42, 45, 51, 52, 53, 54, 55, 56, 58, 59, 61, 62, 64, 66, 67, 68, 69, 70, 71, 72, 73, 81, 82, 83, 84, IS39, CP6108) | β-globin | Roche, Pleasanton, CA, USA |
| SPOCCS III-(2) | Same as SPOCCS III-(1) |  |  |  |  |  |  |
| SPOCCS III-(3) | Same as SPOCCS III-(1) |  |  |  |  |  |  |
| SPOCCS III-(4) | Same as SPOCCS III-(1) |  |  |  |  |  |  |
| SPOCCS III-(5) | Same as SPOCCS III-(1) |  |  |  |  |  |  |
| POCST study-(1) | Sansure HPV genotyping kit | DNA | Target amplification, with real time fluorescence detection | E6/E7/L1 | Separate identification for 15 HPV genotypes: HPV16, 18, 31, 33, 35, 39, 45, 51, 52, 53, 56, 58, 59, 66, 68) | β-globin | Sansure, Changsha, China |
| POCST study-(2) | Same as POCST study-(1) |  |  |  |  |  |  |

**
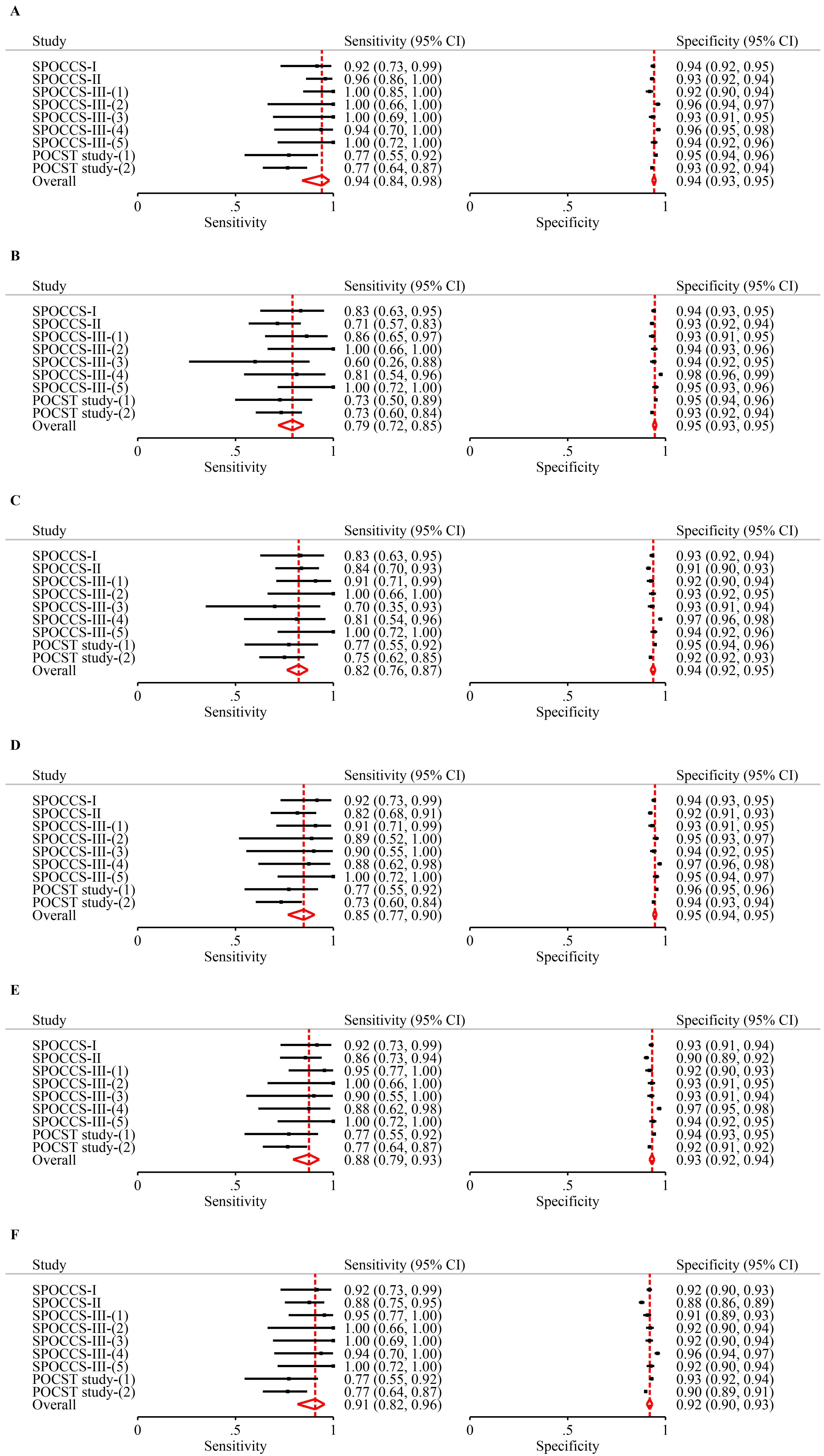
**

**Supplementary Figure S1. Forest plots of the sensitivity and specificity of primary HPV screening with various triage strategies for CIN2+ detection.** **(A)** Forest plot of the sensitivity and specificity of primary HPV screening with HPV16/18 genotyping combined with cytological triage for CIN2+ detection. **(B)** Forest plot of the sensitivity and specificity of primary HPV screening with HPV16/18/58/52 genotyping triage for CIN2+ detection. **(C)** Forest plot of the sensitivity and specificity of primary HPV screening with HPV16/18/58/52/33 genotyping triage for CIN2+ detection. **(D)** Forest plot of the sensitivity and specificity of primary HPV screening with HPV16/18/58/33/31 genotyping triage for CIN2+ detection. **(E)** Forest plot of the sensitivity and specificity of primary HPV screening with HPV16/18/58/52/33/31 genotyping triage for CIN2+ detection. **(F)** Forest plot of the sensitivity and specificity of primary HPV screening with HPV16/18/58/52/33/31/39/51 genotyping triage for CIN2+ detection. The diamonds indicate pooled sensitivity or specificity from the bivariate random-effects model.

Abbreviations: CI, confidence interval; CIN2+, cervical intraepithelial neoplasia grade 2 or higher; HPV, human papillomavirus; POCST study, Point of Care Screening and Treatment for Cervical Cancer Study; SPOCCS, Shanxi Province Cervical Cancer Screening Study.

**
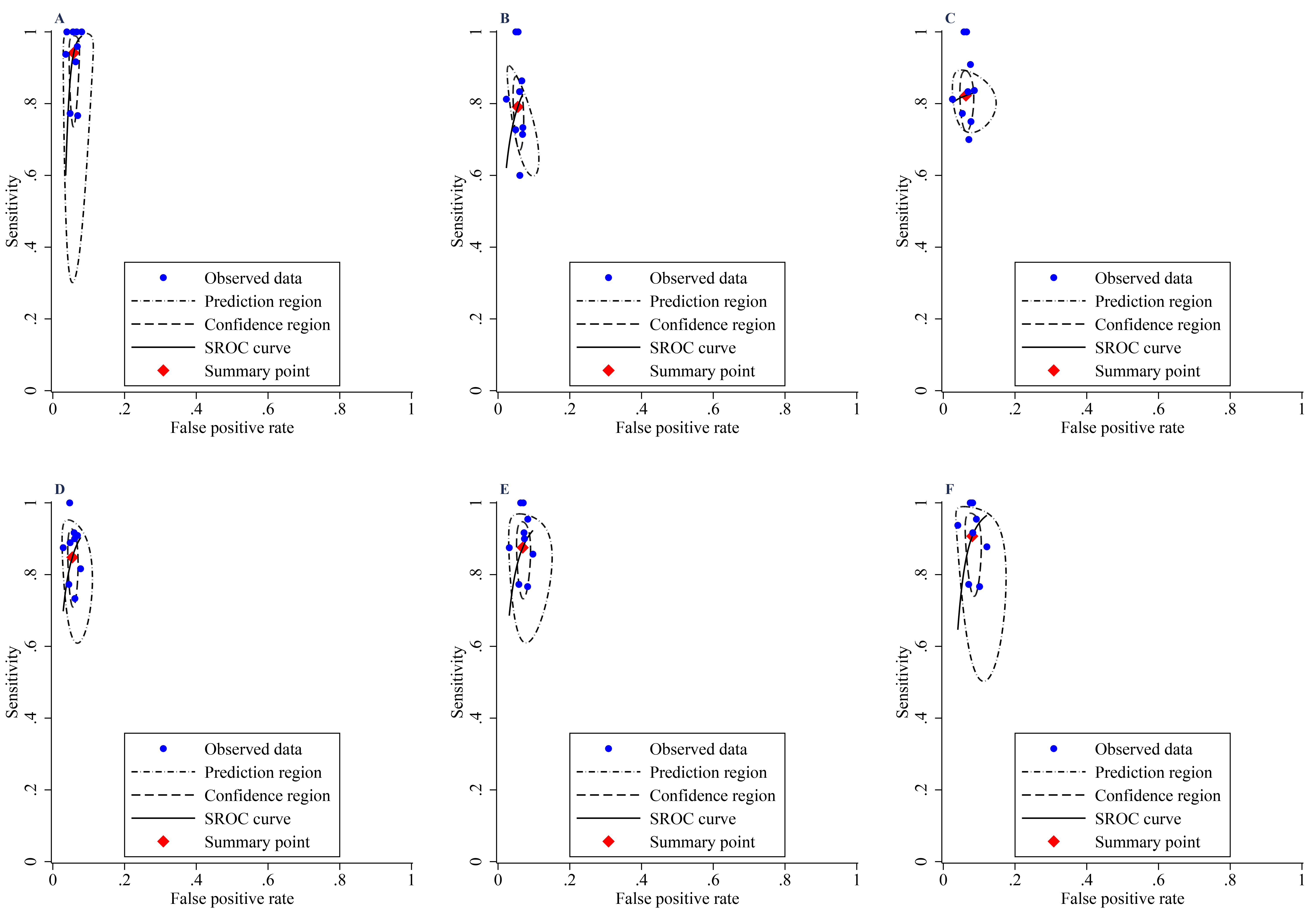
**

**Supplementary Figure S2. Summary of receiver operating characteristic (SROC) curves for primary HPV screening with various triage strategies for CIN2+ detection.** **(A)** SROC curve for primary HPV screening with HPV16/18 genotyping combined with cytological triage for CIN2+ detection. **(B)** SROC curve for primary HPV screening with HPV16/18/58/52 genotyping triage for CIN2+ detection. **(C)** SROC curve for primary HPV screening with HPV16/18/58/52/33 genotyping triage for CIN2+ detection. **(D)** SROC curve for primary HPV screening with HPV16/18/58/33/31 genotyping triage for CIN2+ detection. **(E)** SROC curve for primary HPV screening with HPV16/18/58/52/33/31 genotyping triage for CIN2+ detection. **(F)** SROC curve for primary HPV screening with HPV16/18/58/52/33/31/39/51 genotyping triage for CIN2+ detection.

Abbreviations: CIN2+, cervical intraepithelial neoplasia grade 2 or higher; HPV, human papillomavirus.

**Supplementary Table S2. Diagnostic performance of primary HPV screening with various triage strategies for CIN3+ and CIN2+ detection among women aged ≤45**

| **Triage Strategies** | **Positivity%**  **(No./ Total No.)** | ***P* value** | **Pooled value (95% CI), %** | | **Relative ratio (95% CI)** | | | |
| --- | --- | --- | --- | --- | --- | --- | --- | --- |
|  |  |  | **Sensitivity** | **Specificity** | **Sensitivity ratio** | ***P* value** | **Specificity ratio** | ***P* value** |
| **Detection of CIN3+ (n=37)** |  |  |  |  |  |  |  |  |
| HPV16/18 genotyping in conjunction with cytology (ASC-US threshold) | 7.07 (552/7810) | Ref | 97.30 (83.15-99.62) | 93.36 (92.79-93.89) | Ref | NA | Ref | NA |
| HPV16/18/58/52 genotyping | 6.54 (511/7810) | 0.02 | 92.27 (77.36-97.66) | 94.10 (92.86-95.14) | 0.95 (0.85-1.06) | 0.37 | 1.01 (0.99-1.02) | 0.55 |
| HPV16/18/58/52/33 genotyping | 7.27 (568/7810) | 0.36 | 92.23 (77.20-97.65) | 93.41 (91.96-94.62) | 0.95 (0.85-1.06) | 0.37 | 1.00 (0.98-1.02) | 0.83 |
| HPV16/18/58/33/31 genotyping | 6.13 (479/7810) | < 0.001 | 94.55 (80.56-98.64) | 94.31 (93.26-95.20) | 0.98 (0.88-1.07) | 0.57 | 1.01 (0.99-1.02) | 0.37 |
| HPV16/18/58/52/33/31 genotyping | 7.84 (612/7810) | < 0.001 | 97.43 (81.31-99.70) | 92.88 (91.40-94.13) | 1.00 (0.93-1.08) | 0.99 | 0.99 (0.97-1.01) | 0.44 |
| HPV16/18/58/52/33/31/39/51 genotyping | 9.10 (711/7810) | < 0.001 | 98.71 (30.45-99.99) | 91.80 (90.47-92.97) | 1.02 (0.93-1.12) | 0.79 | 0.98 (0.96-1.00) | 0.06 |
| **Detection of CIN2+ (n=83)** |  |  |  |  |  |  |  |  |
| HPV16/18 genotyping in conjunction with cytology (ASC-US threshold) | 7.07 (552/7810) | Ref | 91.50 (83.17-95.91) | 94.03 (92.89-95.00) | Ref | NA | Ref | NA |
| HPV16/18/58/52 genotyping | 6.54 (511/7810) | 0.02 | 81.93 (71.85-88.96) | 94.48 (93.34-95.44) | 0.90 (0.79-1.01) | 0.08 | 1.00 (0.99-1.02) | 0.56 |
| HPV16/18/58/52/33 genotyping | 7.27 (568/7810) | 0.36 | 84.47 (74.49-91.01) | 93.81 (92.45-94.93) | 0.92 (0.82-1.04) | 0.18 | 1.00 (0.98-1.01) | 0.79 |
| HPV16/18/58/33/31 genotyping | 6.13 (479/7810) | < 0.001 | 88.06 (78.95-93.55) | 94.73 (93.82-95.51) | 0.96 (0.87-1.07) | 0.47 | 1.01 (0.99-1.02) | 0.32 |
| HPV16/18/58/52/33/31 genotyping | 7.84 (612/7810) | < 0.001 | 89.29 (80.23-94.48) | 93.27 (91.89-94.42) | 0.98 (0.88-1.08) | 0.58 | 0.99 (0.97-1.01) | 0.37 |
| HPV16/18/58/52/33/31/39/51 genotyping | 9.10 (711/7810) | < 0.001 | 90.50 (81.67-95.32) | 92.19 (90.89-93.31) | 0.99 (0.90-1.09) | 0.83 | 0.98 (0.96-0.99) | 0.04 |

Abbreviations: AUSROC, areas under the receiver operating characteristic curves; CIN3+, cervical intraepithelial neoplasia grade 3 or higher; ASC-US, atypical squamous cells of undetermined significance; CIN2+, cervical intraepithelial neoplasia grade 2 or higher.

**Supplementary Table S3. Diagnostic performance of primary HPV screening with various triage strategies for CIN3+ and CIN2+ detection among women aged >45**

| **Triage Strategies** | **Positivity%**  **(No./ Total No.)** | ***P* value** | **Pooled value (95% CI), %** | | **Relative ratio (95% CI)** | | | |
| --- | --- | --- | --- | --- | --- | --- | --- | --- |
|  |  |  | **Sensitivity** | **Specificity** | **Sensitivity ratio** | ***P* value** | **Specificity ratio** | ***P* value** |
| **Detection of CIN3+ (n=76)** |  |  |  |  |  |  |  |  |
| HPV16/18 genotyping in conjunction with cytology (ASC-US threshold) | 7.59 (696/9172) | Ref | 95.08 (73.48-99.26) | 94.03 (92.71-95.11) | Ref | NA | Ref | NA |
| HPV16/18/58/52 genotyping | 7.48 (686/9172) | 0.67 | 81.46 (64.01-91.56) | 94.25 (92.79-95.43) | 0.86 (0.71-1.04) | 0.12 | 1.00 (0.98-1.02) | 0.81 |
| HPV16/18/58/52/33 genotyping | 8.56 (785/9172) | < 0.001 | 84.68 (70.06-92.88) | 93.33 (91.71-94.65) | 0.89 (0.75-1.05) | 0.18 | 0.99 (0.97-1.01) | 0.47 |
| HPV16/18/58/33/31 genotyping | 7.46 (684/9172) | 0.59 | 89.82 (72.72-96.69) | 94.24 (92.74-95.45) | 0.94 (0.81-1.10) | 0.47 | 1.00 (0.98-1.02) | 0.81 |
| HPV16/18/58/52/33/31 genotyping | 9.20 (844/9172) | < 0.001 | 93.72 (73.14-98.79) | 92.80 (91.25-94.09) | 0.98 (0.85-1.14) | 0.83 | 0.99 (0.97-1.01) | 0.20 |
| HPV16/18/58/52/33/31/39/51 genotyping | 10.98 (1007/9172) | < 0.001 | 94.92 (73.97-99.19) | 91.48 (89.59-93.05) | 1.00 (0.87-1.14) | 0.99 | 0.97 (0.95-0.99) | 0.02 |
| **Detection of CIN2+ (n=140)** |  |  |  |  |  |  |  |  |
| HPV16/18 genotyping in conjunction with cytology (ASC-US threshold) | 7.59 (696/9172) | Ref | 96.42 (73.76-99.61) | 94.69 (93.38-95.76) | Ref | NA | Ref | NA |
| HPV16/18/58/52 genotyping | 7.48 (686/9172) | 0.67 | 80.50 (66.69-89.49) | 94.94 (93.43-96.11) | 0.83 (0.71-0.98) | 0.04 | 1.00 (0.98-1.02) | 0.79 |
| HPV16/18/58/52/33 genotyping | 8.56 (785/9172) | < 0.001 | 83.90 (71.90-91.39) | 93.99 (92.36-95.29) | 0.87 (0.75-1.00) | 0.07 | 0.99 (0.97-1.01) | 0.47 |
| HPV16/18/58/33/31 genotyping | 7.46 (684/9172) | 0.59 | 87.11 (71.31-94.84) | 94.89 (93.40-96.06) | 0.90 (0.77-1.05) | 0.19 | 1.00 (0.98-1.02) | 0.83 |
| HPV16/18/58/52/33/31 genotyping | 9.20 (844/9172) | < 0.001 | 89.71 (74.36-96.32) | 93.41 (91.86-94.69) | 0.93 (0.81-1.07) | 0.31 | 0.99 (0.97-1.01) | 0.18 |
| HPV16/18/58/52/33/31/39/51 genotyping | 10.98 (1007/9172) | < 0.001 | 94.00 (71.79-98.98) | 92.19 (90.29-93.74) | 0.97 (0.85-1.11) | 0.70 | 0.97 (0.95-0.99) | 0.02 |

Abbreviations: AUSROC, areas under the receiver operating characteristic curves; CIN3+, cervical intraepithelial neoplasia grade 3 or higher; ASC-US, atypical squamous cells of undetermined significance; CIN2+, cervical intraepithelial neoplasia grade 2 or higher.

**
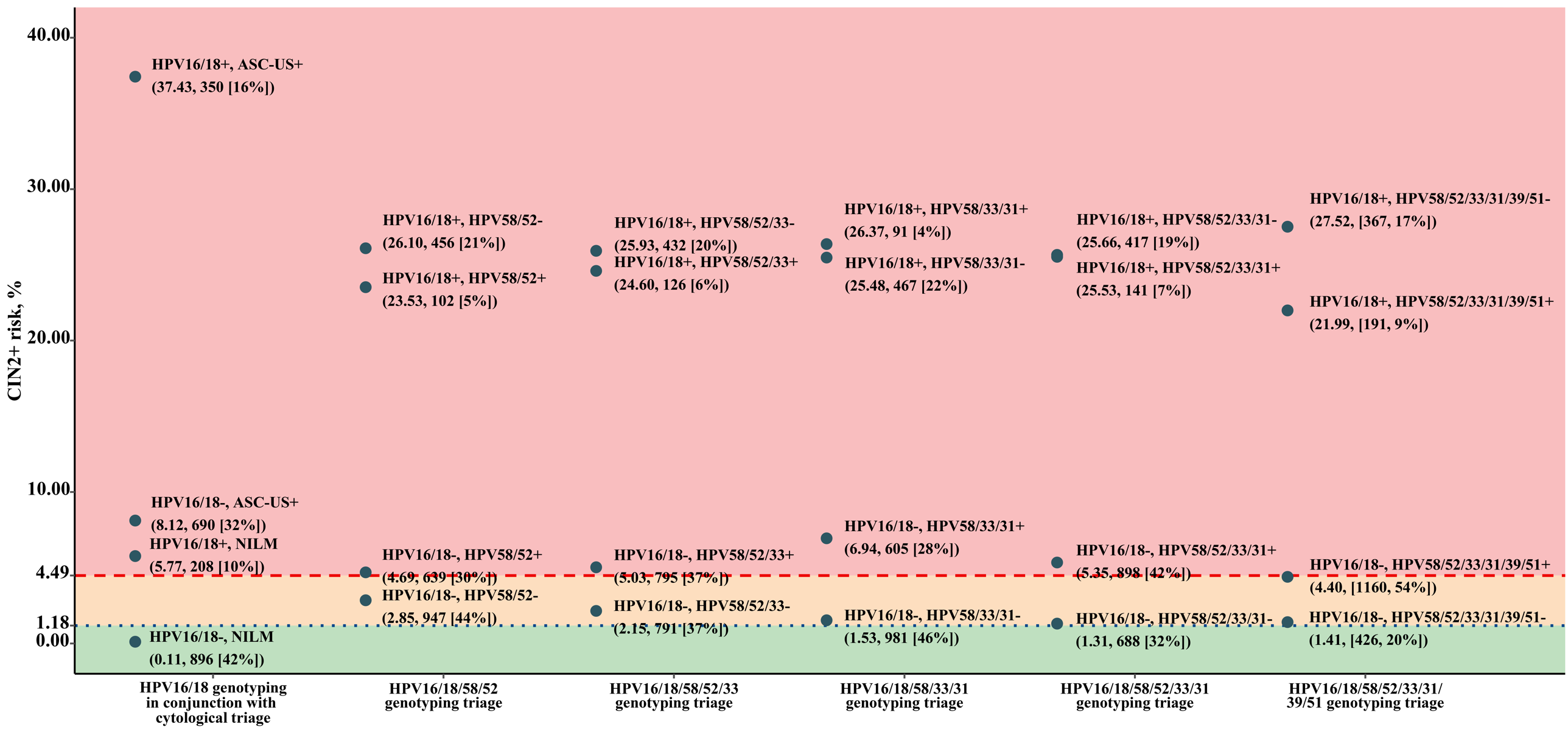
**

**Supplementary Figure S3.** Risk of CIN2+ against internal risk benchmarks according to various triage testing results among high-risk HPV-positive women. The risk of CIN2+ for combinations of HPV16/18 partial genotyping with cytological testing and expanded HPV genotyping alone is plotted on the y-axis and indicated in parentheses. The number and corresponding percentage of women for specific testing result is shown in square brackets. The dotted blue line indicated the 1-year return threshold (HPV-positive and negative for intraepithelial lesion or malignancy [NILM], 1.18%). The dashed red line indicated the colposcopy referral threshold (HPV-positive and atypical squamous cells of undetermined significance [ASC-US], 4.49%). Women with risk falling into the yellow zone were identified as needing one-year return testing. Women with risk falling into the green zone were identified as needing conservative surveillance.

Abbreviations: CIN2+, cervical intraepithelial neoplasia grade 2 or higher; HPV, human papillomavirus.
